# Complete Genome Sequence of Helicobacter pylori Strain GD63, Isolated from a Vietnamese Patient with a Gastric Ulcer

**DOI:** 10.1128/MRA.01412-18

**Published:** 2019-02-21

**Authors:** Thien-Phuc Nguyen-Hoang, Trang Nguyen Hoa, Tan-Huy Nguyen, Stephen Baker, Motiur Rahman, Thuy-Vy Nguyen

**Affiliations:** a Department of Genetics, Faculty of Biology and Biotechnology, University of Science, VNU-HCM, Ho Chi Minh City, Vietnam; b Oxford University Clinical Research Unit, Ho Chi Minh City, Vietnam; c Centre for Tropical Medicine and Global Health, Nuffield Department of Clinical Medicine, Oxford University, Oxford, United Kingdom; d Cancer Research Laboratory, University of Science, VNU-HCM, Ho Chi Minh City, Vietnam; e Khoa Thuong Biotechnology Company, Ho Chi Minh City, Vietnam; Broad Institute of MIT and Harvard University

## Abstract

We present here the first complete genome sequence of Helicobacter pylori strain GD63, isolated from a 72-year-old male Vietnamese patient with a chronic gastric ulcer. The genome consists of a 1.6-Mbp chromosome and an 8.9-kbp plasmid. The strain is *cag* pathogenicity island (*cag*PAI) and VacA positive and belongs to the hpEAsia lineage.

## Abstract (precis)

We present here the first complete genome sequence of Helicobacter pylori strain GD63, isolated from a 72-year-old male Vietnamese patient with a chronic gastric ulcer. The genome consists of a 1.6-Mbp chromosome and an 8.9-kbp plasmid.

## ANNOUNCEMENT

Helicobacter pylori, a Gram-negative bacterium, persistently colonizes half of the population worldwide and is a risk factor for developing a spectrum of gastric pathologies. Its bacterial genomes are diverse among strains from not only different geographic regions but also within the same geographical area (1, 2). The high genetic diversity contributes to variations in the outcomes of infection (3). In Vietnam, the incidence rate of gastric cancer is moderate, although the prevalence of H. pylori infection is high (i.e., 65% to 75% seroprevalence in urban and rural populations) (4, 5). Although many complete and draft genome sequences of H. pylori from different populations have been reported, data on complete genomes from Vietnamese strains are lacking. Here, we present the first complete genome sequence of an H. pylori strain isolated from a gastric ulcer patient in Vietnam.

H. pylori GD63 was isolated from a 72-year-old male patient with a history of gastric pain. A single gastric ulcer was identified in endoscopic examination, and the strain was isolated through gastric biopsy specimen culture on Colombia blood agar medium containing 7.5% sheep blood, 0.4% IsoVitaleX (BD, USA), and 0.4% Dent selective supplement (Oxoid, UK) under microaerophilic conditions at 37°C for 4 to 7 days. The identity of the strain was confirmed by matrix-assisted laser desorption ionization–time of flight (MALDI-TOF; Bruker, Germany) analysis. A single colony was subcultured, and the genomic DNA was extracted using a DNA minikit (Qiagen, Germany). DNA sequencing was conducted using the Illumina MiSeq platform with V3 chemistry (300-bp paired-end reads; Illumina, San Diego, CA) and a Nanopore MinION device with ligation sequencing kit (SQK-LSK108) and an R9.4 ﬂow cell (Oxford Nanopore Technologies, UK). The Illumina reads were trimmed with Trimmomatic (version 0.36) (6), and the Nanopore reads were collected through base calling using the MinKNOW software. We obtained 172,833 high-quality MinION reads and 412,747 MiSeq trimmed reads for the strain. The MinION data were assembled by Unicycler (version 0.4.4) (7). The two resulting circular contigs were polished with the Illumina reads using Pilon (version 1.22) (8), and the sequence of the bridge regions was confirmed by PCR sequencing. The mean coverages of the genome and plasmid were 180× ± 29× and 1,407× ± 146×, respectively. The NCBI Prokaryotic Genome Annotation Pipeline (version 4.6), with default parameters, was used for annotation (9).

The GD63 assembly consists of a circular chromosome of 1,581,994 bp (38.8% GC content) and a plasmid of 8,975 bp (33.4% GC content). The chromosome contains 1,516 coding sequences (CDSs), 3 copies of rRNA genes (5S, 16S, and 23S), 36 coding regions of tRNAs, 2 noncoding RNAs (ncRNAs), and 1 transfer-messenger RNA (tmRNA). GD63 belongs to the hsp EAsia lineage, as determined by multilocus sequence typing (MLST) (10). GD63 contains a complete *cag* pathogenicity island (*cag*PAI) region carrying East Asian-type *cagA* (3,546 bp) and *vacA* (3,966 bp), has the s1m2 genotype, and lacks insertion sequence (IS) elements.

The plasmid of GD63 carries 8 CDSs, including a hypothetical protein. The GD63 plasmid was highly similar (93% identity and 99% coverage) to the pUM196 plasmid (GenBank accession no. KM583819). It encodes a replicase and contains mobilization genes (*mobABCD*), a putative type II toxin/antitoxin system, and putative microcin synthesis and secretion genes (*mccABC*).

### Data availability.

The sequences of the chromosome and plasmid are deposited in GenBank under accession no. CP031558 and CP031559, respectively. Raw data are available in the Sequence Read Archive (SRA) under accession no. SRX5093065 and SRX5093718.
